# High Expression Level of α2-3-Linked Sialic Acids on Salivary Glycoproteins of Breastfeeding Women May Help to Protect Them from Avian Influenza Virus Infection

**DOI:** 10.3390/molecules27134285

**Published:** 2022-07-03

**Authors:** Li Ding, Yimin Cheng, Wei Guo, Siyue Sun, Xiangqin Chen, Tiantian Zhang, Hongwei Cheng, Jiayue Hao, Yunhua Lu, Xiurong Wang, Zheng Li

**Affiliations:** 1 Laboratory for Functional Glycomics, College of Life Sciences, Northwest University, Xi’an 710069, China; liding@nwu.edu.cn (L.D.); sunsiyue@stumail.nwu.edu.cn (S.S.); chenxiangqin@stumail.nwu.edu.cn (X.C.); 202032666@stumail.nwu.edu.cn (T.Z.); 202133269@stumail.nwu.edu.cn (H.C.); haojiayue@stumail.nwu.edu.cn (J.H.); 201932005@stumail.nwu.edu.cn (Y.L.); 2 Department of Obstetrics and Gynecology, Xi’an Shiyou University, Xi’an 710065, China; ymcheng@xsyu.edu.cn; 3 Department of Obstetrics and Gynecology, Shaanxi Provincial People’s Hospital, Xi’an 710068, China; viking226@163.com; 4 National Key Laboratory of Veterinary Biotechnology, Harbin Veterinary Research Institute, Chinese Academy of Agricultural Science, Harbin 150069, China; wangxiurong@caas.cn

**Keywords:** breastfeeding women, saliva, sialic acids (Sia), avian influenza virus (AIV), glycoprotein

## Abstract

Terminal sialic acids (Sia) on soluble glycoprotein of saliva play an important role in the clearance of influenza virus. The aim of this study is to investigate the alteration of sialylation on the salivary proteins of women during the lactation period and its effect on the saliva binding ability to virus. In total, 210 saliva samples from postpartum women with and without breastfeeding were collected, and the expression level of α2-3/6-linked Sia on the whole salivary proteins and specific glycoproteins of IgA and MUC5B from different groups were tested and verified using lectin microarray, blotting analysis and ELISA based method. The H1N1 vaccine and three strains of Avian influenza virus (AIV) were used for the saliva binding assay. Results showed that the variation in salivary expression level of α2-3-linked Sia was much more obvious than the α2-6-linked Sia, which was up-regulated significantly in the breastfeeding groups compared to the non-breastfeeding groups at the same postpartum stage. Furthermore, the binding abilities of salivary glycoproteins to AIV strains and H1N1 vaccine were increased in breastfeeding groups accordingly. This finding adds new evidence for the maternal benefit of breastfeeding and provides new thinking to protect postpartum women from AIV infection.

## 1. Introduction

Avian influenza virus (AIVs) can cause severe respiratory illness and has always been a threat to the life of people worldwide [1]. Furthermore, breastfeeding women are the most concerned population in addition to pregnant women in the past influenza pandemics due to their particular and important physiological statement [2]. During the lactation period, the mammary glands can be induced by the high expression level of prolactin to secrete sufficient maternal milk that is unusually rich in glycosylated proteins to provide the neonatal protection [3,4]. Besides which, salivary glands can also be regulated by hormones and secrete plenty of glycoproteins that are important in the maintenance of mucosal protection [5]. It has been reported that the glycosylation of salivary proteins can be changed with different physiological and pathological conditions [6,7,8]. However, little is known about the variation in salivary glycosylation during the special period of lactation and its effect on the salivary proteins binding ability to AIVs. 

The oral cavity is an entry site where pathogens from outside can come into contact with the host cells, and human saliva is known as the most important of biological fluids, which can provide the first line of immune defense to resist virus attachment [9,10]. Now, up to more than a thousand proteins have been found in human saliva, but only a dozen of them are in abundance, such as Muc5B (mucin 5B), Muc7 (mucin 7), secretoryimmunoglobulinA (SIgA), cysteine-rich glycoprotein 340 (gp-340), prolactin inducible protein (PIP), and so on [11,12]. Most of these salivary proteins are highly glycosylated, and the carbohydrate part cantake up to 80% of the total protein weight (e.g., MUC5B). The glycans on salivary proteins are usually composed of carbohydrates such as glucose, galactose, mannose, fucose, amino sugars, et al. Among them, sialic acids (Sia) are the most typical ones, which are a diverse family of 9-carbon monosaccharides with N-acetylneuraminic acid (Neu5Ac) as their basic molecular structure and often added to the penultimate sugar—usually galactose (Gal) or its derivatives in α2-3 or α2-6 linkage [13]. This kind of Sia-containing structure usually appears on the outermost terminal of glycoconjugates and play an important role in the cell to cell signaling and microbe-host recognition, and immune regulation [14,15,16]. In particular, the Siaα2,3/6Gal-linked glycans can be used as receptors for the hemagglutinin (HA) and substrates for neuraminidase (NA) of influenza virus, by which the virus can bind to or release from the host epithelial cells and perform their infection process [17,18]. Different species of influenza viruses usually recognize different linkages of sialic acids, avian influenza viruses predominantly bind to α2,3-linked Sia, whereas human influenza viruses prefer to bind to α2,6-linked Sia on host cells [19]. The successful anti-influenza drugs, such as Oseltamivir, Zanamivir et al., are structural analogs that imitate sialosides and can be used as competitive substrate inhibitors of NA to resist the influenza viruses infection [20,21]. As the natural barrier, it has been proved that the soluble salivary proteins glycosylated with terminal α2-3/6-linked Sia can neutralize and inhibit influenza viruses in this way efficiently [22,23,24]. And the protein sialylation level in saliva has been expected to be used as an indicator to estimate the individual susceptibility to influenza virus [25].

As one of the most important glycosylation, the sialylation level on salivary proteins have been shown that can be varied according to different ages and sexes, and changed in elderly individuals with autoimmune diseases, chronic diseases, cancers and so on [26,27]. During female gestation, along with the fluctuation of hormone and the repression of the immune system, pregnancy-associated changes of sialylation have also been demonstrated both in serum and salivary proteins before [28]. After delivery, the hormones such as estrogens and progesterone start to resume, while the prolactin remains at a high level, especially in the early stage of postpartum and is quite different between postpartum women with and without breastfeeding. Therefore, the sialylation level on the glycoproteins of saliva during this period, which can directly involve in the anti-virus activity, is of great interest, to be explicit. 

In this paper, the individual saliva samples from postpartum women with and without breastfeeding were collected and the alteration of terminal α2,3/6-linked Sia on salivary glycoproteins between these two groups were investigated and compared at different postpartum stages, and the binding affinities of salivary protein to the influenza virus were assessed by using the H1N1 vaccine and different AIV strains of H5N1-CK (A/Chicken/Guangxi/4/2009), H5N1-DK (A/Duck/Guangdong/17/2008) and H9N2-DK (A/Duck/Guangdong/S-7-134/2004). The results of this study will help us to further understand the influence of breastfeeding on the maternal risk of AIV infection, which will give a new thinking to improve the health statement of postpartum women. 

## 2. Materials and Methods

### 2.1. StudyApprovaland Participants

The collection of human whole saliva for this study was approved by the Human Ethics Committees of Shaanxi Provincial People’s Hospital, Xi’an Shiyou University and Northwest University, China. A total of 210 individual saliva samples from breastfeeding or non-breastfeeding postpartum women were collected, and healthy women in non-pregnant and non-breastfeeding statementwere used as control.

The participants that were recruited were in different periods of postpartum: first-period (FP, within six monthspostpartum); second-period (SP, from 7th to 12th month postpartum); and third-period (TP, from 13th to 24th month postpartum). Subjects were divided into seven groups (Supplementary Table S1), including the control group of healthy women without pregnancy and breastfeeding (HN) and the FP, SP, TP groups of breastfeeding women (FP-B, SP-B, TP-B) and non-breastfeeding women (FP-NB, SP-NB, TP-NB). Each group consisted of 30 samples.

### 2.2. Whole Saliva Sample Collection and Treatment

About 1mL of saliva sample was collected from each participant according to the protocol described previously [26,27,28]. The saliva sample was centrifuged at 12,000 rpm, 4 °C for 20 min to get rid of the insoluble materials. The supernatant was then collected and added with the cocktail inhibitor of protease (Sigma-Aldrich, St. Louis, MO, USA) to avoid protein degradation. Each of 200 μL saliva sample, from different individuals in the same group, were blended and the protein concentration of each blended group of samples was quantified by the BCA (bicinchoninicacid) assay (listed in Supplemental Table S1), and labeled with Cy3 (cyanine3fluorescent dye) (GE Healthcare, Buckinghamshire, UK).

### 2.3. Lectin Microarrays and Data Analysis

According to the previous protocol [26,27,28], the lectin microarray was produced by using 37 lectins with different binding preferences to N- and O-linked glycans. Each lectin was spotted in triplicate in one block, and with three blocks on one slide. The slide was blocked with 2% BSA in 1× PBS (0.01 mol/L phosphate buffer containing 0.15 mol/L NaCl, pH 7.4) (*w*/*v*, pH 7.4) and washed before use. Then, 5 μg of Cy3-labeled salivary proteins from each group diluted in 0.75 mL of hybridization buffer (2% BSA, 500 mM glycine and 0.1% Tween-20 in PBS, pH 7.4) was applied to each block of the slide, and incubated in the dark at 37 °C for 3h with gentle rotation. After being washed with 1× PBST (0.2% Tween 20 in 0.01 mol/L phosphate buffercontaining 0.15 mol/L NaCl, pH 7.4) and 1× PBS (0.01 mol/L phosphate buffer containing 0.15 mol/L NaCl, pH 7.4), and dried by centrifugation, the slides were scanned using a Genepix 4000B confocal scanner (Axon Instruments Inc., Union City, CA, USA), and analyzed at 532 nm by the Genepix 3.0 software (version 6.0, Axon Instruments). For data analysis, the background signal was substracted, and the median value of the triplicate data for each lectin was selected and normalized to the sum median of all the lectins in one block. The average and standard deviation (SD) of the normalized medians for each lectin in different blocks tested for the same sample were calculated, and the difference between two groups was evaluated by the *p* value. 

### 2.4. SDS-PAGE and Lectin Blotting Analysis

Equal amounts of salivary proteins from each group were separated by 10% sodium dodecylsulfate polyacrylamide gel electrophoresis (SDS-PAGE), and then stained either with alkaline silver or with Periodic Acid-Schiff staining (PAS) and Coomassie Brilliant Blue R250(CBB). For lectin blotting analysis, after SDS-PAGE, the proteins in each gel were transferred to a 0.45 µmpolyvinylidene fluoride (PVDF) membrane at a constant current of 300 mA for 45–80 min according to their molecular weight, blocked with Carbo-Free Solution (Vector, Burlingame, CA, USA) and then incubated with the cyanine5fluorescent dye (Cy5)-labeled maackia amurensis lectin II (MAL-II) and sambucus nigra lectin (SNA) at 4 °C in the dark overnight. After that, the membranes were washed with 1× TBST (150 mM NaCl, 10 mM Tris-HCl, 0.05% *v*/*v* Tween 20, pH 7.5) and 1× TBS (150 mM NaCl, 10 mM Tris-HCl) respectively, and scanned by using the FluorImager (Storm 840, Molecular Dynamics Inc., Sunnyvale, CA, USA). The intensity of the binding band on the acquired image was further analyzed by the ImageJ software (NIH), and the relative fluorescence intensity (RFI) ofeachbandfor different groups was calculated and compared to the control group of HN.

### 2.5. Assessment of Salivary Proteins Binding Ability to AIVs

All the AIV strains used in this study were kindly presented from Harbin Veterinary Research Institute, China. The H1N1 influenza A vaccine (Split Virion, inactivated) was purchased from the company of Sinovac Biotech Ltd., Beijing. According to the method described in the previous literature [26,27], the viral proteins were extracted with a mixture of ethanol and ether by being thoroughly shaken. After static placement, the ether phase was discarded, and the proteins in the remaining liquid were dried and labeled with Cy5 dye. For the binding ability assay, an equal amount of 50μg proteins from each group were run on the 10% SDS-PAGE electrophoresis, transferred to 0.45 µm PVDF membrane, and then incubated with the Cy5-labeled viral proteins.The image scan and the analysis of band intensity were the same as that described above.

### 2.6. Western Blot Analysis

Equal amount of 20 μg salivary proteins from different groups were applied to the 10% SDS-PAGE and transferred from gels to the PVDF membrane as described in 2.4. After being blocked with 5% skimmed milk in TBST for 1 h, the membrane was incubated with primary antibody of mouse monoclonal anti-human MUC5B (1:1000 in TBST, Abcam, Ab105460) or rabbit monoclonal anti-human immunoglobulin A (IgA) (1:1000 in TBST, Abcam, Ab124716) at 4 °C overnight. The membrane was then washed three times with TBST and incubated with the horseradish peroxidase (HRP)-conjugated secondary antibody for 1 h at room temperature. The goat anti-mouse Immunoglobulin G heavy and light chains (IgG H&L) for MUC5B (1:5000 in TBST; Abcam, Ab205719) and the goat anti-rabbit IgG H&L for IgA (1:5000 in TBST, Abcam, Ab205718) were used, respectively. After being washed three times with TBST, the membranes were finally detected by using the chemiluminescent HRP substrate (Beyotime, Haimen, China) and scanned with the Tannon 5200 Imaging System (Tanon, Shanghai, China).

### 2.7. ELISA Based Lectin Binding Assay

For immobilization, 100 μL of 5 μg/mL MUC5B antibody (Abcam, Ab105460) or IgA antibody (Abcam, Ab124716) diluted in the coating buffer (0.1 M carbonate/bicarbonate, pH 9.4) was added to each well of the 96-well plate and incubated at 4 °C overnight. The wells used as the blank control were added with 100 μL of coating buffer without any antibody. The next day, the plate was washed three times with PBST and blocked with carbo-free solution at 37 °C for 1 h on the shaker. Then, the plate was washed again and 50 μg of salivary proteins (1 μg/μL) from each group was added to the well in triplicate and incubated at 37 °C for 1 h. After the plate being washed, 100 μL of the biotinylated SNA or MAL-II (Vector Laboratories Inc, Burlingame, CA, USA), which was 1:3000 or 1:200 diluted in carbon-free solution, was applied to each well and incubated at 37 °C for another 1 h. Next, the plate was washed again and 100 μL of streptavidin-HRP (Vector Laboratories) with 1:2000 dilution in carbon-free was added to the well and incubated at 37 °C for 50 min. The HRP signal was finally detected by 3,3′,5,5′-tetramethylbenzidine (TMB) substrate (Beyotime, Haimen, China) and the absorbance of 450 nm for each well was read by the microplate reader (Bio-Rad Laboratories, Hercules, CA, USA).

### 2.8. Statistical Analysis

Statistical analysis was performed by the SPSS software (version 20.0), and the *p* value between two different groups was acquired by Mann–Whitney test. The difference was considered statistically significant if the *p* value was <0.05.

## 3. Results

### 3.1. Relative Expression Levels of Terminal α2-3/6-Linked Siain Saliva Groups of Postpartum Women with and without Breastfeeding

**Figure 1:**
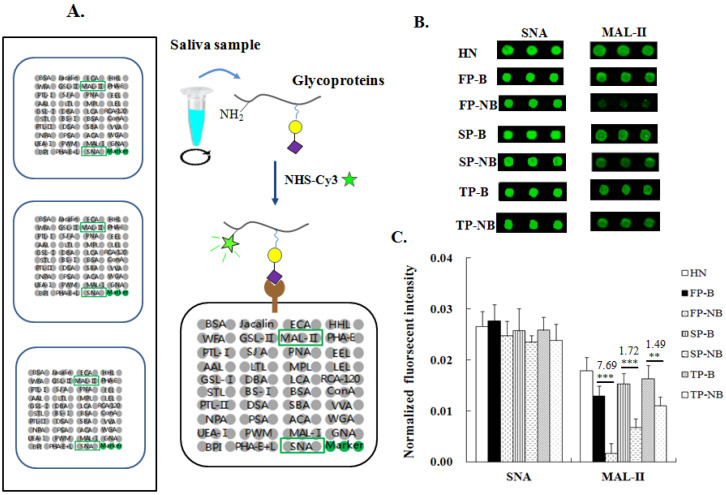
Comparison of the terminal α2-3/6-linked Sia expression level on salivary proteins of postpartum women with and without breastfeeding. (**A**) The label of saliva samples with Cy3 fluorescent dye and the layout of lectin microarray. (**B**) Fluorescent images of SNA detected forα2-6-linked Sia and MAL-II detected for α2-3-linked Sia from the lectin microarrays used for different groups. (**C**) Normalized fluorescent intensity (NFI) of SNA and MAL-II binding to salivary proteins of different groups. FP-B: first-period of postpartum women with breastfeeding; FP-NB: first-period of postpartum women without breastfeeding; SP-B: second-period of postpartum women with breastfeeding; SP-NB: second-period of postpartum women without breastfeeding; TP-B: third-period of postpartum women with breastfeeding; TP-NB: third-period of postpartum women without breastfeeding. Each sample was applied to three repeated slides, the standard deviation (SD) of NFIs for each lectin from all the repeated blocks was calculated. Data are shown as mean ± SD, Statistical significance between two different groups was analyzed by Student’s *t*-test and indicated by the *p*-value. ** *p* < 0.01; *** *p* < 0.001.

In total, 210 saliva samples were collected from breastfeeding (B) and non-breastfeeding (NB) women in the first, second, third period (FP, SP,TP) of postpartum, and healthy women without pregnancy, and breastfeeding were used as the control group (Supplementary Table S1). The expression level of terminal α2-3 and α2-6-linked Sia on salivary proteins of different groups were detected by the specific lectin of SNA and MAL-II using lectin microarray (Figure 1A). The fluorescent images of Cy3-labeled salivary proteins binding to SNA or MAL-II on the lectin microarray were shown in Figure 1B. The normalized fluorescent intensities (NFIs) of SNA and MAL-II for different groups were listed in Supplementary Table S1 and further analyzed in Figure 1C. 

From the result, it can be seen that the salivary expression level of α2-6-linked Sia was a little bit higher in the breastfeeding women than that of the non-breastfeeding ones at the same postpartum stage, but the discrepancy between these two groups was not too much (Figure 1C). While for the α2-3-linked Sia, its expression level was found to be significantly up-regulated in the breastfeeding groups of FP-B, SP-B, TP-B compared to non-breastfeeding groups of FP-NB, SP-NB, TP-NB, especially for the women within six months postpartum (fold change = 7.67, *p* < 0.001). The discrepancy was reduced along with the extension of postpartum time.

### 3.2. Analysis for the Salivary Proteins and the Variation in Terminal α2-3/6-Linked Sia Expression Level on Glycoproteins of Different Groups

**Figure 2:**
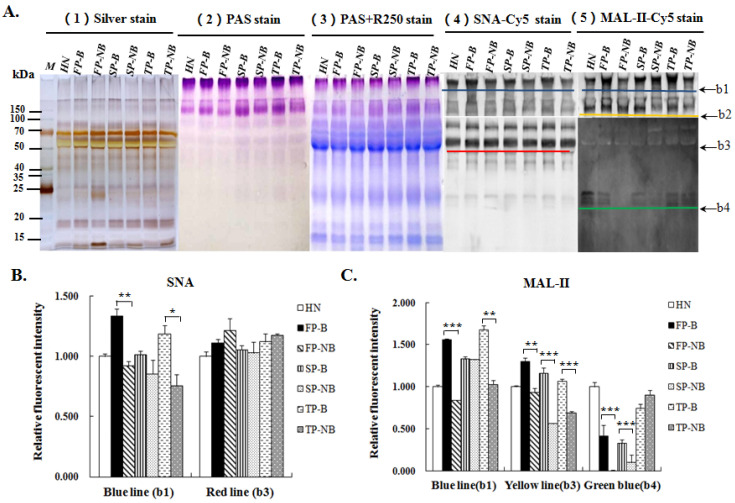
Analysis of the salivary proteins profiles and the variation in terminal α2-3/6-linked Sia expression level on glycoproteins of different groups. (**A**) Salivary proteins and glycoproteins from different groups were shown by the silver stain (1), PAS stain(1) and the combine of PAS and Coomassie Brilliant Blue R-250 stains (3), and the salivary proteins glycosylated with terminal α2-3/6-linked sialic acids were detected by the lectin blotting analysis of Cy5-labled SNA (4) and MAL-II (5). (**B**) Relative fluorescence intensities (RFIs) of the binding bands of salivary proteins to Cy5-labled SNA. (**C**) RFIs of the binding bands of salivary proteins to Cy5-labled MAL-II. Data shown are mean ± SD, * *p* < 0.05; ** *p* < 0.01; *** *p* < 0.001.

Salivary proteins with and without glycosylation were shown by using different staining methods after SDS-PAGE (Figure 2A). Results showed that saliva samples from all the groups were composed of proteins with the similar molecular weight. PAS staining showed that there were two bands of proteins, which are heavily glycosylated, one of the band was shown in the stacking gel (band 1, b1) and the other one was around 150 kD (band 2, b2). Besides, there were another two glycosylated protein bands, which can be seen more apparently by the sensitive fluorescent detection of Cy5-labeled SNA (band 3, b3) and MAL-II (band 4, b4) below 100 kD.

The expression level of terminal Siaα2-3/6Gal on salivary proteins of different groups were further investigated by the Lectin blotting analysis (Figure 2A(4,5)), and the samples treated with α2-3 or α2-3,6,8 sialidase were used as the blank control (Supplementary Figure S1). The Relative fluorescence intensities (RFIs) of the SNA and MAL-II binding bands between groups of women with and without breastfeeding were analyzed and shown in Figure 2B,C. The lectin binding image of SNA (Figure 2A(4)) showed that the salivary proteins glycosylated with terminal Siaα2-6Gal mainly appeared on the proteins of band 1 in the stacking gel and band 3 around 50 kD. The expression levels of Siaα2-6Gal on the proteins of band 3 showed no significant difference between women with and without breastfeeding. While for band 1, the expression level of Siaα2-6Gal in the saliva of the breastfeeding group was obviously increased compared with that of non-breastfeeding group at the same stage (Figure 2B). As to the lectin blotting of MAL-II, there were three distinct binding bands (band 1 in the stacking gel, band 2 around 150 kD, band 4 around 25 kD) to be observed. Apparent discrepancy of RFIs can be found for all of these three bands between breastfeeding groups and non-breastfeeding groups, the expression levels of Siaα2-3Gal on the salivary proteins of these bands were obviously higher in the former groups than that in the latter groups (Figure 2C).

### 3.3. Comparison of Salivary Proteins Binding Ability to AIVs between Postpartum Women with and without Breastfeeding

**Figure 3:**
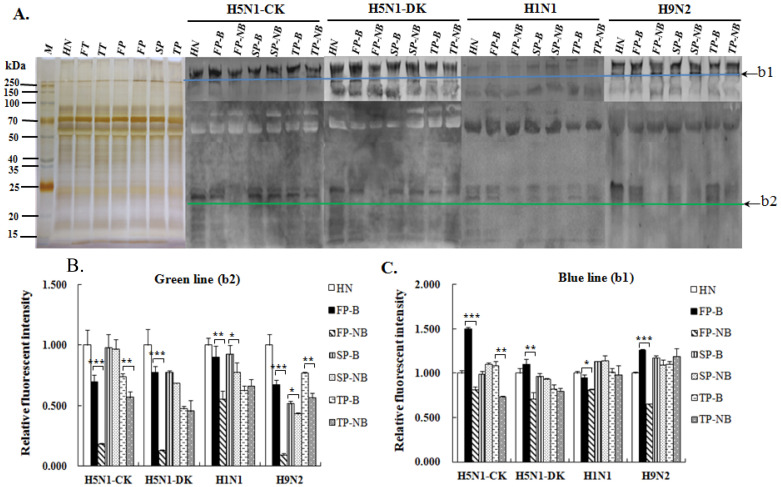
Assessment of the binding ability of salivary proteins from different groups to the AIV strains and the H1N1 influenza A vaccine. (**A**) The binding profiles of salivaryproteins to the H1N1 vaccine and the AIV strains of H5N1-CK (A/Chicken/Guangxi/4/2009), H5N1-DK (A/Duck/Guangdong/17/2008) and H9N2- DK (A/Duck/Guangdong/S-7-134/2004). (**B**) RFIs of the binding band shown at around 25 kD for different saliva groups. (**C**) RFIs of the binding band shown in the stacking gel for different saliva groups. Data shown are mean ± SD, * *p* < 0.05; ** *p* < 0.01; *** *p* < 0.001.

The binding abilities of salivary proteins to influenza virus were assessed using the inactivated H1N1 virus (H1N1 vaccine) and three AIV strains (H5N1-CK, H5N1-DK, H9N2). An equal amount of salivary proteins from different groups were separated by the SDS-PAGE and then blotted with the Cy5-labeled proteins of virus. From the binding images (Figure 3), there were two bands of proteins, which can bind strongly to all of the virus, one of the band presented in the stacking gel and the other band appeared at about 25 kD. The RFIs of the binding bands for different saliva groups were compared (Figure 3B,C). For the AIV strains, significant differences can be seen between the groups of women with and without breastfeeding at both of these two bands. Furthermore, the virus-binding intensities of salivary proteins from breastfeeding groups were much stronger than that from non-breastfeeding women, especially within six months postpartum. These results were consistent with the binding affinity of salivary proteins to MAL-II. As to H1N1 virus, the binding intensities of these two bands to different saliva groups were not obviously varied, as found in the AIVs, and that may be due to the HA of H1N1 preferring to bind to α2,6-linked SA receptor, so the change of α2,3-linked SA level in saliva could not affect its binding affinity too much. Besides, there was also a strong binding band at around 70 kD for H1N1 and H9N2 strains and a week binding band at around 150 kD for H5N1-DK, H1N1 and H9N2 strains. For the band around 150 kD, the binding intensity was weak and its variation in different groups was identical to the band in the stacking gel. As to the binding intensity around 70 kD, its variation in different groups was obviously not too much.

### 3.4. Expression Level of IgA and MUC5B in the Saliva of Postpartum Women with and without Breastfeeding

IgA and MUC5B are known as the primary glycoproteins in human saliva, which can exert high anti-influenza activity through their sialylated glycochains [23,29]. From the binding profile of lectin blotting analysis, it can be seen that the protein bands shown in the stacking gel and at around 50 kD, 25 kD were identical with the molecular weight of MUC5B and IgA heavy and light chains, respectively. Therefore, the protein expression level of MUC5B and IgA and the variation in Sia were further investigated in different groups. 

**Figure 4:**
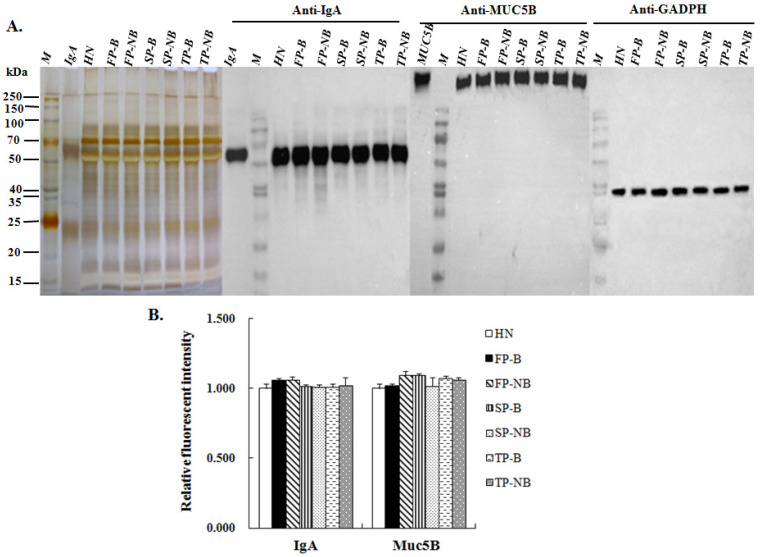
Analysis ofthe MUC5B and IgA expression level in different saliva groups. (**A**) The binding images of salivaryproteins corresponding to human anti-MUC5B or anti-IgA antibody, glyceraldehyde-3-phosphate dehydrogenase (GAPDH) was used as the loading control. (**B**) RFIs of the binding bands of salivary proteins corresponding to human anti-MUC5B or anti-IgA antibody. Data shown are mean ± SD, statistical notations and group abbreviations are same as shown in Figure 1.

After SDS-PAGE, the salivary proteins on each gel were incubated with the human anti-IgA or anti-MUC5B antibody, respectively. The binding band of salivary proteins corresponding to anti-IgA antibody and anti-MUC5B antibody appeared at around 50 kD and in the stacking gel as expected (Figure 4A). The RFIs of the corresponding bands between groups of women with and without breastfeeding were compared and shown in Figure 4B. The result showed that the protein expression level of IgA or MUC5B were not varied during the lactating period, and there was no significant difference between groups of breastfeeding and non-breastfeeding women. 

### 3.5. Expression Level of Terminal α2-3/6-Linked Sia Expressed on IgA and MUC5B in Different Saliva Groups

**Figure 5:**
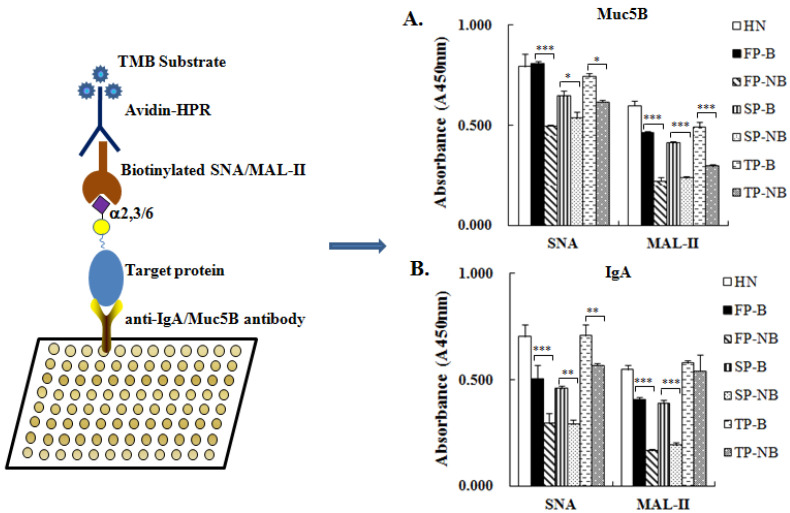
Comparison of theterminal α2-3/6-linked Sia expression level on MUC5B and IgA between saliva groups of women with and without breastfeeding. Salivary MUC5B or IgA was captured by the anti-human MUC5B or anti-human IgA antibody on 96-well plate, and the expression level of terminal α2-3/6-linked Sia, which was recognized by the biotinylated SNA/MAL-II and finally detected by the Avidin-HPR system. The absorbance at 450 nm was read for the analysis of SNA/MAL-II binding to IgA (**A**) and MUC5B (**B**) from different groups. Data shown are mean ± SD, statistical notations and group abbreviations are same as shown in Figure 1.

Enzyme-linked immunosorbent assay (ELISA) based lectin binding assay was applied to evaluate the level of α2-3/6-linked sialic acids expressed on IgA and MUC5B of different groups. The IgA and MUC5B from different salivary groups were firstly captured by the human anti-IgA or anti-MUC5B antibodies that were immobilized on the 96-well plate. Then the Siaα2-3/6Gal structures on the specific protein of IgA and MUC5B were recognized by the biotinylated SNA or MAL-II and detected by the HPR labeled avidin. The results showed that both the expression levels of Siaα2-3/6Gal on MUC5B (Figure 5A) and IgA (Figure 5B) were up-regulated in the saliva of postpartum breastfeeding women compared with non-breastfeeding women. The increased expression level of Siaα2-3/6Gal on MUC5B of the breastfeeding women was consistent with the result of the lectin blotting analysis for the binding band that appeared in the stacking gel. As for IgA, the improved expression level of Siaα2-3Gal in breastfeeding women coincided with the lectin binding profiles analyzed for the band around 25 kD, which was identical with the molecular weight of IgA light chain. But for the expression of Siaα2-6Gal on IgA, its increased trend in the breastfeeding groups was not in agreement with the lectin blotting of SNA for the band at a little above 50 kD, where the heavy chain of IgA may present. This may be caused by the binding of other glycoproteins with the similar molecular weight in saliva.

## 4. Discussion

Pregnancy and lactation are the particular physiological periods of women with dramatic fluctuation of hormones. It is reported that along with the hormone alteration, the innate immunity response to respiratory virus infection was affected [30,31]. The morbidity and mortality for pregnant women were obviously higher than the general population during the past influenza pandemics [2]. After delivery, the prolactin in breastfeeding women remains at a high level compared with non-breastfeeding women. However, their susceptibility to influenza virus has not yet been clear.

Considering the importance of α2-3/6-linked sialic acids involved in the anti-influenza virus activity, their expression level on the salivary proteins of women with and without breastfeeding were investigated in this study, and the associated salivary glycoprotein binding abilities to different strains of influenza virus were accessed. From the result of lectin microarray, the salivary expression level of α2-6-linked sialic acids between lactating women and non-lactating women was not varied too much on the whole level. While for the expression level of α2-3-linked sialic acids, the discrepancy between these two kinds of groups was much more significant, which is apparently higher in the breastfeeding groups than the non-breastfeeding groups at the same postpartum stage. This result was further verified by the lectin blotting analysis, the binding profile of salivary proteins to MAL-II showed that there are apparently three bands of proteins glycosylated with α2-3-linked sialic acids. From the RFI data, it can be seen that the expression level of α2-3-linked sialic acids on the proteins of all these three bands was much higher in the saliva of breastfeeding women and the binding abilities of salivary glycoproteins to AIV strains of H5N1-CK, H5N1-DK, H9N2 were found to be increased in breastfeeding groups accordingly. From the fluorescent binding images of salivary glycoproteins to different AIV strains, there are two binding bands with varied RFI between breastfeeding and non-breastfeeding groups. The one that appeared in the stacking gel was in accordance with the high molecular weight of salivary MUC5B, and the other band around 25 kD coincided with the molecular weight of IgA light chain. Given that both of the MUC5B and IgA are primary glycoproteins in human saliva with high anti-influenza activity, their protein and sialylation levels were further tested using Western blotting analysis and ELISA based MAL-II/SNA binding assay. The results showed that the protein level of MUC5B or IgA was almost the same among different saliva groups. As for the sialylation level, a significant discrepancy can be seen between the groups of women with and without breastfeeding, and the variation trend of α2-3 sialylation on the protein of MUC5B or IgA in different groups was consistent with that observed on the whole saliva level, which were significantly increased in the groups of breastfeeding women in comparison to the non-breastfeeding groups. These results suggested that the high expression of α2-3 sialylation in the whole saliva samples of lactating women mainly come from the glycosylation level rather than the protein level.

It is known that the terminal sialic acids on glycoproteins play an important role in the regulation of immune effects, and their expression can be varied according to different physiological and pathological conditions [14,15,16]. We have previously demonstrated that the expression of α2-3-linked sialic acids on salivary proteins was decreased in pregnant women, which was associated with their high susceptibility to AIV [28]. While in this study, the expression of α2-3-linked sialic acids was found to be increased significant in breastfeeding women under the high level of prolactin hormone. These results were accordant with the different influenza infection risk of women during pregnancy and postpartum periods that was reported in a recent study [2]. The different variation trend of α2-3 sialylation could be due to the physiological changes of hormones during pregnancy and lactation periods and their different influence on the immune system. It has been reported that the progesterone hormone has immunoinhibitory effects during pregnancy, whereas prolactin has the opposite effects of immunostimulatory [32,33]. Moreover, prolactin has also been proved to be involved in the regulation of glycosyltransferases for the synthesis of lactose in human milk [34]. The saliva glands and the glycosylation of their secretory products could also be modulated by hormones in this way [35], and the underling mechanism is of interest to be studied in the future.

It is well established that breastfeeding can provide numerous health benefits, not only for infants, but also for mothers [36]. The advantages of breastfeeding to mothers have been illustrated, including the improved glucose metabolism, the reduction in hypertension and hyperlipidemia, and the lower risks of breast and ovarian cancer, heart disease, type 2 diabetes et al. [37,38]. Here, our study further indicated that breastfeeding could be helpful to protect women from AIV infection by increasing the expression level of α2-3 linked sialic acids on their salivary proteins. This finding adds more evidence for the maternal benefit of breastfeeding, and provides a new support to encourage mothers to choose breastfeeding during postpartum.

In this study, the changes in sialylation level were detected and verified by using a different method, and investigated both on the whole saliva level and on the specific glycoproteins. However, there still some limitations. First, the sample size for each group is not too large. More samples need to be recruited to improve the accuracy. Second, the anti-influenza activity of saliva samples was not accessed by using the influenza virus directly, the saliva inhibition assays of hemagglutination and neutralization will make the result more meaningful. Finally, the mechanism of prolactin influence on the α2-3 sialylation of salivary proteins for breastfeeding women was not referred to in this study, and as such, requires further research. 

## Data Availability

Not applicable.

## Supplementary Materials

The following supporting information can be downloaded at: https://www.mdpi.com/article/10.3390/molecules27134285/s1, Figure S1: The binding profiles of SAN (A) and MAL-II (B) to salivary proteins treated with sialidase; Table S1: The demographic characteristics of the healthy control and postpartum women with and without breastfeeding.

## Abbreviations

AIV: Avian influenza virus; Sia, sialic acids; Gal, galactose; MUC5B, mucin5B; MUC7, mucin 7; HA, humagglutinin; NA, neuraminidase; IgG H&L, immunoglobulin G heavy and light chains; IgA, immunoglobulin A; SD, standard deviation; BCA, Bicinchoninicacid; Cy3/5, cyanine 3/5 fluorescent dye; SDS-PAGE, sodium dodecylsulfate polyacrylamide gel electrophoresis; PVDF, polyvinylidene fluoride; MAL-II, maackia amurensis lectin II; SNA, sambucus nigra lectin; RFI, relative fluorescence intensity; HRP, horseradish peroxidase; TMB, 3,3′,5,5′-tetramethylbenzidine; GAPDH, glyceraldehyde-3-phosphate dehydrogenase; ELISA, enzyme-linked immunosorbent assay; FP-B/NB, first-period of postpartum women with/without breastfeeding;SP-B/NB, second-period of postpartum women with/without breastfeeding; TP-B/NB, third-period of postpartum women with/without breastfeeding.
