# Supplementary material for: High Expression Level of α2-3-Linked Sialic Acids on Salivary Glycoproteins of Breastfeeding Women May Help to Protect Them from Avian Influenza Virus Infection
Source: Molecules. 2022 Jul 3;27(13):4285. doi: 10.3390/molecules27134285 (PMC9268398; doi:10.3390/molecules27134285)
Supplement: Supplementary file 1 [file molecules-27-04285-s001.zip › molecules-1676993-supplementary.pdf]

Supporting information for

High Expression Level of α2-3-Linked Sialic Acids on Salivary Glycoproteins of Breastfeeding Women May Help to Protect Them from Avian Influenza Virus Infection

Li Ding [1], Yimin Cheng [2],Wei Guo [3], Siyue Sun [1], Xiangqin Chen [1],Tiantian Zhang [1], Hongwei Cheng [1], Jiayue Hao [1], Yunhua Lu [1], Xiurong Wang [4] and Zheng Li [1,*]

1 Laboratory for Functional Glycomics, College of Life Sciences, Northwest University, Xi'an 710069, China; liding@nwu.edu.cn (L.D.); sunsiyue@stumail.nwu.edu.cn (S.S.); chenxiangqin@stumail.nwu.edu.cn (X.C.); 202032666@stumail.nwu.edu.cn (T.Z.); 202133269@stumail.nwu.edu.cn (H.C.); haojiayue@stumail.nwu.edu.cn (J.H.); 201932005@stumail.nwu.edu.cn (Y.L.)
2 Department of Obstetrics and Gynecology, Xi'an Shiyou University, Xi'an 710065 , China; ymcheng@xsyu.edu.cn
3 Department of Obstetrics and Gynecology, Shaanxi Provincial People's Hospital, Xi'an 710068, China; viking226@163.com
4 National Key Laboratory of Veterinary Biotechnology, Harbin Veterinary Research Institute, Chinese Academy of Agricultural Science, Harbin 150069, China; wangxiurong@caas.cn
* Correspondence: zhengli@nwu.edu.cn

Table S1. The demographic characteristics of the healthy control and postpartum women with and without breastfeeding.

Parameter		FP-B	FP-NB	SP-B	SP-NB	TP-B	TP-NB
Sample number(n)		30	15	30	30	30	30
Age, years, median, SD		29.2±2.5	29.4±3.5	30.1±4.5	30.6±4.6	30.2±3.9	31.1±3.7
Bradford assay	OD_{595}	0.914	0.875	0.771	0.870	0.899	0.875
	PC (mg/mL)	0.905	0.860	0.739	0.854	0.888	0.860
Lectin	Specificity	NFI±SD					
SNA	Siaα2-6Gal/GalNAc	0.0227 ±0.0038	0.0216 ±0.0068	0.0231 ±0.0029	0.0247 ±0.0018	0.0234 ±0.0048	0.0253 ±0.0046

		Fold change					
		FP-NB /FP-B	FP-B /FP-NB	SP-NB /SP-B	SP-B /SP-NB	TP-NB /TP-B	TP-B /TP-NB
		0.95	1.05	0.94	1.07	0.93	1.08
Lectin	Specificity	NFI±SD					
MAL-II	Siaα2-3Galβ1-4Glc (NAc)/Glc, Siaα2-3Gal, Siaα2-3GalNAc	0.0100 ±0.0013	0.0013 ±0.0013	0.0107 ±0.0022	0.0062 ±0.0021	0.0130 ±0.0026	0.0087 ±0.0013
		Fold change					
		FP-NB /FP-B	FP-B /FP-NB	SP-NB /SP-B	SP-B /SP-NB	TP-NB /TP-B	TP-B /TP-NB
		0.13***	7.69***	0.58***	1.72***	0.67**	1.49**

FP-B: first-period of postpartum women with breastfeeding; FP-NB: first-period of postpartum women without breastfeeding; SP-B: second-period of postpartum women with breastfeeding; SP-NB: second-period of postpartum women without breastfeeding; TP-B: third-period of postpartrum women with breastfeeding; TP-NB: third-period of postpartrum women without breastfeeding. ** $p < 0.01$, and *** $p < 0.001$

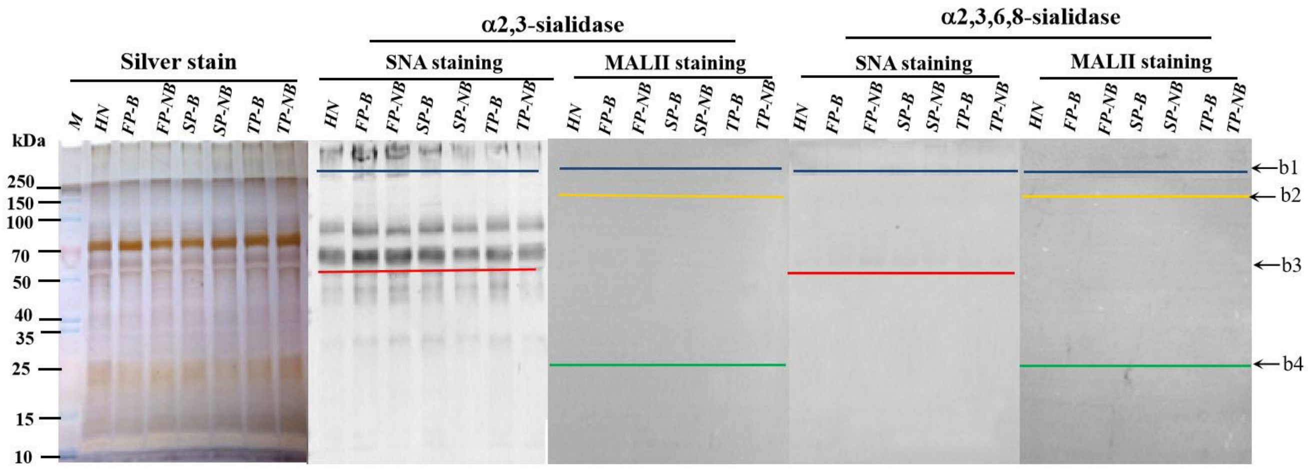

Figure S1 The binding profiles of SAN (A) or MAL-II (B) to salivary proteins treated with sialidase

FP-B: first-period of postpartum women with breastfeeding; FP-NB: first-period of postpartum women without breastfeeding; SP-B: second-period of postpartum women with breastfeeding; SP-NB: second-period of postpartum women without breastfeeding; TP-B: third-period of postpartrum women with breastfeeding; TP-NB: third-period of postpartrum women without breastfeeding.
